# Knowledge and awareness on vector-borne diseases: a pending subject for the Spanish society?

**DOI:** 10.23938/ASSN.1080

**Published:** 2024-08-30

**Authors:** Moisés Gonzálvez, María del Mar Asensio, Clara Muñoz-Hernández, Rocío Ruiz de Ybáñez

**Affiliations:** 1 Universidad de Murcia Campus de Excelencia Internacional Regional “Campus Mare Nostrum” Facultad de Veterinaria Departamento de Sanidad Animal Murcia Spain; 2 Universidad de Córdoba Departamento de Sanidad Animal Grupo de Investigación en Sanidad Animal y Zoonosis (GISAZ) Córdoba Spain; 3 Instituto de Investigación en Recursos Cinegéticos IREC (CSIC-UCLM-JCCM) Grupo Sanidad y Biotecnología (SaBio) Ciudad Real Spain

**Keywords:** Vector Borne Diseases, Spain, Knowledge, Perception, Surveys and Questionnaires, Enfermedades Transmitidas por Vectores, España, Conocimiento, Percepción, Encuestas

## Abstract

**Background:**

To evaluate the level of knowledge and risk perception regarding vector-borne diseases in the Spanish society and identify the risk factors associated with the level of understanding.

**Methodology:**

An online survey was conducted between January and May 2021, targeting Spanish adults. The survey consisted of 11 questions assessing knowledge and risk perception related to vector-borne diseases. Sociodemographic variables predicting the outcome variable (objective level of knowledge, determined by correctly naming two vector-borne diseases) were analyzed using multiple logistic regression. The outcome variable was then compared with the self-reported knowledge declared by the participants (kappa coefficient, k).

**Results:**

Out of the 1,646 respondents who completed the survey, 72.2% were female and 59.8% were >40 years old). Additionally, 87.3% showed a high risk perception and 61.7% understood what is a vector; however, only 36.3% had an objective level of knowledge (*k*= 0.353). The variables being >40 years (p <0.0001), having a monthly income >1,500 € (p <0.0001), and owning pets (p= 0.0423) were positively related with an objective level of knowledge on vector-borne diseases (adjusted effects).

**Conclusions:**

Both knowledge and perception of the risk on vector-borne diseases need to be strengthened in Spain, especially in regions where vector-borne diseases are endemic, emerging, or re-emerging. These preliminary results underscore the necessity of enhancing institutional efforts to build a robust knowledge base within the Spanish society, extending beyond scientific forums.

## RESUMEN

**Fundamento:**

Evaluar el nivel de conocimiento y percepción del riesgo de la sociedad española respecto a enfermedades transmitidas por vectores e identificar los factores de riesgo relacionados con su conocimiento.

**Material y métodos:**

Encuesta online con once preguntas sobre el conocimiento y percepción del riesgo de las enfermedades transmitidas por vectores, enviada entre enero y mayo de 2021 a adultos españoles. Mediante regresión logística múltiple se analizaron las variables sociodemográficas predictoras de la variable de resultado (*tener un nivel objetivo de conocimiento*: haber nombrado correctamente dos enfermedades transmitidas por vectores), que se comparó con el conocimiento declarado por los participantes mediante el coeficiente kappa (k).

**Resultados:**

De los 1.646 encuestados (72,2% mujeres, 59,8% con edad >40 años), el 87,3% mostraron elevada percepción de riesgo y el 61,7% declararon tener conocimiento sobre qué es un vector. Sin embargo, solamente el 36,3% demostraron tener un nivel objetivo de conocimiento (*k*= 0,353). Las variables edad >40 años (p <0,0001), ingresos mensuales >1.500€ (p <0,0001) y ser propietario de mascotas (p= 0,0423) se asociaron a tener un nivel objetivo de conocimiento sobre enfermedades transmitidas por vectores (efectos ajustados).

**Conclusiones:**

Tanto el conocimiento como la percepción del riesgo de las enfermedades transmitidas por vectores deben reforzarse en España, especialmente en áreas endémicas, emergentes y reemergentes de enfermedades transmitidas por vectores. Este resultado preliminar confirma la necesidad de mejorar los mecanismos institucionales para promover una sólida base de conocimientos en la sociedad española fuera de los foros científicos.

## INTRODUCTION

Currently, vector-borne pathogens account for over 25% of all infectious diseases, causing millions of human deaths annually. Additionally, they have a significant impact on animal health and the global economy^1^^,^^2^. Although tropical and subtropical regions traditionally support the highest burden of vector-borne diseases (VBDs)^3^, a multifactorial set of factors now influences the global distribution of vectors and, consequently, the pathogens they transmit. Among these factors, climatic change stands out as the primary abiotic factor driving the widespread distribution of VBDs, as it significantly affects the biological cycle of vectors^4^. However, other human-related factors, such as population growth, globalization, changes in land use, and human migration, also contribute to the large-scale spread of VBDs^4^^,^^5^. This complex network of variables influencing exposure risk to vector-borne pathogens highlights the need for a broad, integrated approach to studying these diseases.

European countries bordering the Mediterranean Sea are significant hotspots for emerging and re-emerging VBDs^6^. The Mediterranean basin is particularly vulnerable to changes in VBD epidemiology due to potential variations in climatic conditions in the region^7^. For example, there has been an increase in VBDs in Spain, notably the West Nile and Crimean-Congo hemorrhagic fever viruses, among others^8^^,^^9^. Not only have these pathogens caused infections in animals, but also led to human deaths in the region^10^^,^^11^.

The significance of Spain as a hotspot for VBDs is enhanced by its strategic geographical location, which provides both a favorable climate for vector breeding and key stopover sites for migratory birds^12^. Despite the epidemiological evidence indicating that Spain is a hotspot for VBD affecting animals and humans, a recent study revealed a significant lack of knowledge and awareness about Chagas disease among the Spanish population^13^.

Although social factors directly affect the epidemiology of VBDs, more in-depth studies are needed to assess knowledge and risk perception. This information is key for developing effective prevention and control programs for at-risk populations.

This study aimed to evaluate the level of knowledge and risk perception among the Spanish population on VBDs and identify risk factors that influence the level of knowledge and awareness about these issues.

## MATERIAL AND METHODS

A survey was conducted from January to May 2021 using an online questionnaire randomly distributed by a network of collaborators across all Spanish bioregions. The target population consisted of Spanish volunteers aged 18 and older; non-Spanish respondents were excluded. The questionnaire link was shared via social media, instant messaging apps, and e-mail distribution lists. All respondents were firstly informed about the study´s purpose and asked for their explicit consent to participate voluntarily.

The questionnaire was designed to assess know-ledge and risk perception regarding VBDs and their risk factors. It included questions on general knowledge about vectors, VBDs, and clinical cases in humans and/or animals, as well as opinions on the impact of VBDs on humans, domestic animals and wildlife, and the awareness of politicians, scientists, and the public.

1. Are you aware of what a vector is? Yes / No
2. Do you know that vectors can transmit diseases? Yes / No
3. Are you concerned about VBDs? Yes / No / I don’t know
4. Do you know of any family member or a friend who has been affected by a VBD? Yes / No / I don’t know
5. Do you know of any animal that has been affected by a VBD? Yes / No / I don’t know
6. Do you believe that VBDs have a negative impact on domestic animals? Yes / No / I don’t know
7. Do you believe that VBDs have a negative impact on wild animals? Yes / No / I don’t know
8. Do you believe that VBDs have a negative impact on humans? Yes / No / I don’t know
9. Do you believe scientists are concerned about VBDs? Yes / No / I don’t know
10. Do you believe politicians are concerned about VBDs? Yes / No / I don’t know
11. Do you believe the society is concerned about VBDs? Yes / No / I don’t know

Those respondents who demonstrated some level of knowledge by correctly naming at least two VBDs were classified as having an objective level of knowledge (KN-VBD).

The questionnaire also requested the following information:

- Nationality
- Age (years), categorized as 18-20, 21-30, 31-40, 41-50, 51-65, or >65 years old
- Sex: Male / Female
- Province of residence, framed within the Spanish bioregions (B1-B6)^14^
- Population size of the area were the respondent lives: ≤50,000 / >50,000 inhabitants
- University studies: Yes / No
- Monthly income (in Euro, €), asked as <600 / 601-1,500 / 1,501-2,500 / 2,501-3,500 / 3,501-4,500 / >4 ,500; and re-categorized as ≤1,500 / 1,501-2,500 / >2,500
- Workplace: Indoor (inside a building) / Outdoors / Mixed / Other
- Owner of domestic animals: Yes / No

For all questions, the frequency and percentage (%) of responses in each category was calculated. The association between the response variable KN-VBD (Yes / No) and explanatory categorical variables (individual information) was initially assessed using Pearson’s Chi-squared test or Fisher’s exact test, as appropriate. Variables with p-values <0.10 were selected for inclusion in the multivariate analysis. Collinearity between variables was first assessed using the Cramer’s V coefficient, selecting the variable with the highest biological plausibility; if a correlation coefficient >0.6 and a p-value <0.02 were obtained, the variable with the highest plausibility was selected. Finally, the influence of the selected explanatory variables on objective knowledge about VBDs (KN-VBD) was evaluated using logistic regression models, assuming a binomial data distribution and treating “province” as a random effect. The Akaike Information Criterion was used to select the most accurate model, resulting in the inclusion of age, monthly income, and ownership of domestic animals in the final model. Additionally, to assess reliability between self-reported knowledge on VBDs and actual knowledge (KN-VBD), a Kappa coefficient (k) was calculated and interpreted as slight (0.00-0.20), fair (0.21-0.40), moderate (0.41-0.60), substantial (0.61-0.80), and almost perfect (>0.80)^15^. Statistical significance was set at p <0.05, and all statistical analyses were conducted using the R software^16^.

Furthermore, to identify relationships among the most frequently reported VBDs by respondents, we used VOSviewer^®^ (http://www.vosviewer.com/) to identify clusters of terms extracted from their answers. VOSviewer^®^ is a free software for creating and visualizing semantic networks or term maps from scientific literature^17^^,^^18^. The software employs text mining and clustering functions to analyze the co-occurrence of terms and construct the semantic network^17^ that is divided into thematic clusters, each represented by a different color. The diameter of the colored circles in the network corresponds to the frequency with which each term was cited in the reviewed publications.

## RESULTS

**Figure 1:**
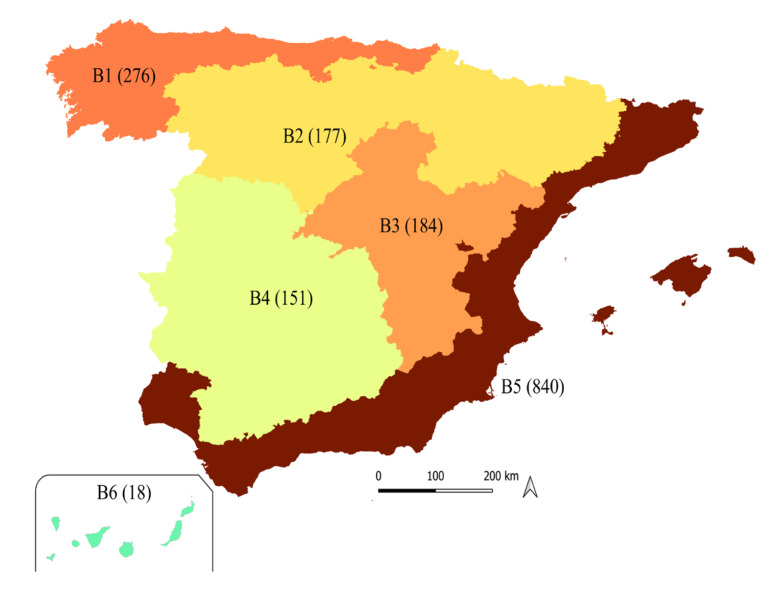
Distribution of respondents’ answers across the six bioregions of Spain.

A total of 1,646 responses were obtained from Spanish participants across all bioregions. The number of respondents ranged from 18 participants in B6 to 840 participants in B5 (Fig. 1).

The response rate was higher among female participants (72.2%) and varied by age, ranging from 3.7% for responders aged 18-20 years to 29.4% for those aged 51-65 years. A larger number of responses was obtained from individuals aged >40 years (n=985; 59.8%). Additionally, 63.9% of the participants owned domestic animals and 85.7% had completed university studies (Table 1).

**Table 1:** Characteristics of the respondents

Variable	n (%)
	N=1,646
Age (years)	
18-20	61 (3.7)
21-30	350 (21.3)
31-40	250 (15.2)
41-50	439 (26.7)
51-65	484 (29.4)
>65	62 (3.7)
Sex	
Female	1,188 (72.2)
Bioregion	
B1	276 (16.7)
B2	177 (10.8)
B3	184 (11.2)
B4	151 (9.2)
B5	840 (51.0)
B6	18 (1.1)
Size of locality residence	
>50,000	835 (50.7)
Level of education	
University	1,410 (85.7)
Monthly income (€)	
<1,500	478 (29.0)
1,501-2,500	538 (32.7)
>2,500	630 (38.3)
Workplace	
Indoors	976 (59.3)
Outdoors	84 (5.1)
Mixed	316 (19.2)
Other	270 (16.4)
Owner of domestic animals	
Yes	1,051 (63.9)

Overall, 597 respondents (36.3%) demonstrated their knowledge by correctly naming at least two VBDs and were classified as KN-VBD. Among all participants, 87.3% expressed concern about VBDs, with 38.8% of them classified as KN-VBD. Similar patterns were observed among respondents who claimed to know what a vector is (61.7%) and the diseases they transmit (94.3%); however, a lower percentage of these respondents were classified as KN-VBD (49.4% and 37.9%, respectively). These discrepancies between the participants´ self-reported knowledge and their actual understanding of VBDs (KN-VBD) were confirmed by a low kappa coefficient (*k =* 0.353), indicating a fair level of agreement.

Twenty-eight per cent respondents reported knowing a family member or friend affected by a VBD and 44.9% reported knowing of domestic animals with VBDs; 35.6% and 39.4% of these respondents were classified as KN-VBD, respectively. Approximately 80-90% of the participants believed that VBDs have negative health implications for domestic animals, wildlife, and humans, with 35-40% of them classified as KN-VBD. Regarding public opinion, the Spanish society perceived that scientists (66.1%) are the most concerned group about VBDs, followed by the general public (21.3%); only 3.6% of respondents considered VBDs a major issue for politicians (Table 2). Similarly, 33.1-42.2% of respondents who expressed concern about scientists, politicians, and general public were classified as KN-VBD. Additionally, participants who chose “*I don’t know*”, tended to have lower percentages of KN-VBD compared to those who answered “*yes*” or “*no*”.

**Table 2:** Frequency of responses of vector-borne disease risk and objective knowledge (KN-VBD) in the Spanish population

Questions	Risk percetion n (%)	KN-VBD n (%)	p-value (χ^2^)
Are you aware of what a vector is?			<0.0001
Yes	1,016 (61.7)	502 (49.4)	
No	630 (38.3)	95 (15.1)	
Do you know that vectors can transmit diseases?			<0.0001
Yes	1,553 (94.3)	589 (37.9)	
No	93 (5.7)	8 (8.6)	
Are you concerned about VBDs?			<0.0001
Yes	1,437 (87.3)	558 (38.8)	
No	75 (4.6)	17 (22.7)	
I don't know	134 (8.1)	22 (16.4)	
Do you know of any family member or a friend who has been affected by a VBD?			0.015
Yes	461 (28)	164 (35.6)	
No	676 (41.1)	290 (42.9)	
I don't know	509 (30.9)	143 (28.1)	
Do you know of any animal that has been affected by a VBD?			0.0003
Yes	739 (44.9)	291 (39.4)	
No	510 (31)	195 (38.2)	
I don't know	397 (24.1)	111 (28.0)	
Do you believe that VBDs have a negative impact on domestic animals?			<0.0001
Yes	1,492 (90.6)	571 (38.3)	
No	27 (1.6)	7 (25.9)	
I don't know	127 (7.7)	19 (15.0)	
Do you believe that VBDs have a negative impact on wild animals?			<0.0001
Yes	1,377 (83.7)	556 (40.4)	
No	43 (2.6)	5 (11.6)	
I don't know	226 (13.7)	36 (15.9)	
Do you believe that VBDs have a negative impact on humans?			<0.0001
Yes	1,517 (92.2)	585 (38.6)	
No	32 (1.9)	4 (12.5)	
I don't know	97 (5.6)	8 (8.2)	
Do you believe scientists are concerned about VBDs?			<0.0001
Yes	1,088 (66.1)	459 (42.2)	
No	154 (9.4)	38 (24.7)	
I don't know	404 (24.5)	100 (24.8)	
Do you believe politicians are concerned about VBDs?			0.579
Yes	59 (3.6)	24 (40.7)	
No	1,371 (83.3)	500 (36.5)	
I don't know	216 (13.1)	73 (33.8)	
Do you believe the society is concerned about VBDs?			<0.0001
Yes	350 (21.3)	116 (33.1)	
No	1,022 (62.1)	406 (39.7)	
I don't know	274 (16.6)	75 (27.4)	
Global	1,646	597 (36.3)	

VBD: vector-borne diseases.

Based on the univariate analysis, three out of eight explanatory variables were statistically associated with the level of knowledge about VBDs (KN-VBD). Specifically, the analysis revealed diffe-rences in KN-VBD across age groups, with respon-dents aged 21-30 years showing the highest level of knowledge (47.4%) and those >65 years the lowest (21.0%). Additionally, the proportion of KN-VBD was positively associated with monthly income and was higher among respondents with domestic animals (38.3%). In contrast, there were no statistical differences between knowledge about VBDs and Spanish bioregions (Table 3).

**Table 3:** Variables associated with objective knowledge about vector-borne diseases (KN-VBD) among the Spanish population (univariate analyses)

Variable	n	KN-VBD n (%)	p-value (χ^2^)
Age (years)			<0.0001
18-20	61	20 (32.8)	
21-30	350	166 (47.4)	
31-40	250	111 (44.4)	
41-50	439	128 (29.2)	
51-65	484	159 (32.9)	
>65	62	13 (21.0)	
Sex			0.616
Male	458	171 (37.3)	
Female	1,188	426 (35.9)	
Bioregion			0.523
B1	276	102 (36.9)	
B2	177	69 (38.9)	
B3	184	73 (39.7)	
B4	151	44 (35.8)	
B5	840	344 (40.9)	
B6	18	7 (38.8)	
Size of locality residence			0.133
≤50,000	811	279 (34.4)	
>50,000	835	318 (38.1)	
Level of education			0.203
No university	236	32 (13.6)	
University	1410	565 (40.2)	
Monthly income (€)			0.0008
≤1,500	478	145 (30.3)	
1,501-2,500	538	192 (35.7)	
>2,500	630	260 (41.7)	
Workplace			0.917
Indoors	976	357 (36.6)	
Outdoors	84	32 (38.1)	
Mixed	316	119 (37.7)	
Other	270	89 (33.0)	
Owner of domestic animals			0.023
Yes	1,051	403 (38.3)	
No	595	194 (32.6)	

The multivariate logistic regression model confirmed that age (<40 years), ownership of domestic animals, and a monthly income >1,500 € were the main factors associated with KN-VBD (Table 4).

**Figure 2:**
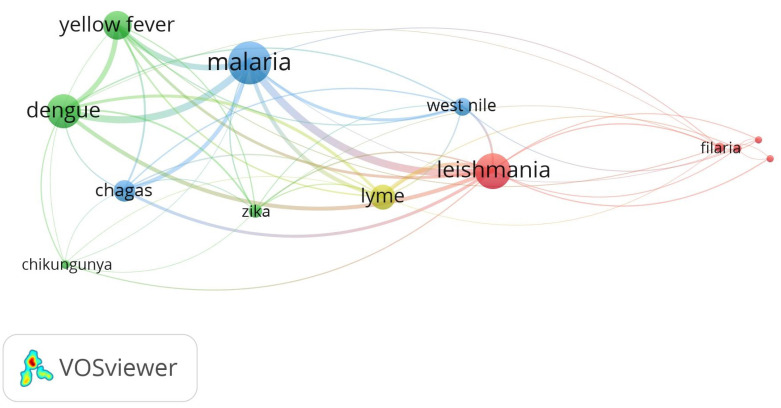
Relationships between pathogens and diseases caused by vector-borne diseases based on respondent terms identified by the semantic network. The network is divided into thematic clusters; blue cluster: *Chagas*, *malaria*, and *West Nile*; green: *yellow fever, dengue,* and *zika*; red: *leishmania* and *filaria*; *Lyme* disease is simultaneously linked to all three clusters. The diameter of the colored circles in the network corresponds to the frequency with which each term was cited in the reviewed publications.

The semantic network analysis conducted using VOSviewer^®^ revealed three predominant clusters. Among these clusters, *malaria*, *Leishmania*, *yellow fever*, and *dengue* were the most frequently mentioned pathogens and diseases. The most common word pairs included *malaria*-*Leishmania*, followed by *malaria-dengue*, *malaria-yellow fever*, and *malaria-Chagas* (Fig. 2).

**Table 4:** Variables associated to the objective knowledge about vector-borne diseases (KN-VBD) among the Spanish population (multivariate logistic regression model)

Variable	n	OR (95% CI)	χ^2^	p-value
Fixed effects				
Age			28.99	<0.0001
<40 years	61	1		
>40 years	85	0.55 (0.45-0.69)		
Monthly income (€)			18.53	0.0001
≤1,500	78	1		
1,501-2,500	38	1.32 (1.01-1.74)		
>2,500	30	1.77 (1.36-2.31)		
Owner of domestic animals			4.12	0.0423
No	95	1		
Yes	1051	1.26 (1.01-1.58)		
Random effect	Variance	Standard deviation		
Province	0.09	0.30		

OR: *odds ratio*; 95% CI: 95% confidence interval.

## DISCUSSION

Knowledge about VBDs among the Spanish population is lower than expected, as KN-VBD weakly correlates with respondents who self- reported understanding *what a vector is* and whether *vectors transmit diseases*. Regarding spatial distribution, our results suggest a homogeneous level of VBD knowledge across Spain. This is in line with the application of similar educational standards throughout the country and the national relevance of analogous endemic VBDs, as seen with *Leishmania infantum*^19^^,^^20^.

Although a small percentage of respondents know of family/friends or domestic animals affected by VBDs, the majority (>80%) are aware that these pathogens can have severe implications for domestic animals, wildlife, and humans. These differences reinforce our findings, indicating that many respondents are concerned about VBDs despite having no direct experience with them. This is consistent with the analyzed semantic network, where, of the most frequently mentioned pathogens by respondents, only two - *Leishmania* and the West Nile virus - are considered endemic and have significant health implications in Spain^11^^,^^19^. Our results suggest that the perception of VBDs among the Spanish society is primarily shaped by external information rather than personal experiences. This highlights the importance of investing in knowledge dissemination in non-scientific areas to maximize its outreach to society.

Overall, responders believe there is significant differences between VBD risk perception by scientists and those held by the general public. This suggests that the Spanish population recognizes the important role researchers play in the study of VBDs. The lowest awareness on VBDs among the studied groups is observed among politicians; only 3.6% of respondents believe they are concerned about VBDs. If this perception is confirmed, the difficulty in establishing preventive and control measures could have negative health implications. This is particularly concerning in the current epidemiological context of VBDs where competent authorities should remain vigilant.

The study reveals that younger respondents (<40 years old) possess a greater level of knowledge about VBDs. This is in line with the results by Sahoo et al.^21^ who reported that younger individuals generally have a higher level of knowledge of VBD compared to older adults. A previous study indicated that younger individuals are more likely to take protective measures against VBDs compared to older people^22^. Study participants who own pets possess greater knowledge about VDBs, which may be linked to the health impact of these diseases in domestic animals, as seen with leishmaniasis or the West Nile virus in dogs and horses, respectively^20^^,^^23^. In contrast, this information contradicts previous scientific data that indicate a general lack of knowledge among pet owners about diseases affecting their animals^24^^,^^25^. Additionally, a higher monthly income (>1,500 €) associates with greater knowledge about VBDs. Previous surveys suggest that lower incomes are linked to a weaker perception of disease risk^26^^,^^27^.

There are some limitations to this study. For instance, the underrepresentation of populations without internet access and the inability to verify the accuracy of the responses may have influenced the results. Regarding the employed methodology, volunteer participation may introduce bias, as respondents who chose to participate may have a higher awareness and knowledge about VBDs compared to those that had access to the questionnaire but chose not complete it^28^. Therefore, these methodological biases should be taken into account when interpreting the results.

The knowledge and risk perception of VBDs among the Spanish population are key issues that need to be strengthened in regions where VBDs are endemic, emerging, or re-emerging. Our results provide a foundation for further research and/or management strategies focused on VBDs, especially those with zoonotic implications. Additionally, educating the general public about VBDs from multiple perspectives, such as detection, prevention, and control, is crucial for building a solid knowledge base. To achieve this, institutional mechanisms should be enhanced, particularly through the dissemination of information about VBDs in non-scientific forums. This includes awareness campaigns on social media and TV, scientific outreach activities, and basic training in schools.
